# Development of a Hopkinson Bar Apparatus for Testing Soft Materials: Application to a Closed-Cell Aluminum Foam

**DOI:** 10.3390/ma9010027

**Published:** 2016-01-05

**Authors:** Marco Peroni, George Solomos, Norbert Babcsan

**Affiliations:** 1 European Commission Joint Research Centre (JRC), IPSC, ELSA, Via E. Fermi 2749, Ispra (VA) 21027, Italy; george.solomos@jrc.ec.europa.eu; 2 Aluinvent Zrt., Ipari Park, Szeles utca 2, Felsőzsolca H-3561, Hungary; norbert.babcsan@aluinvent.com

**Keywords:** Hopkinson bar, dynamic material properties, aluminum foams, high strain-rate, soft-materials, MHPB

## Abstract

An increasing interest in lightweight metallic foams for automotive, aerospace, and other applications has been observed in recent years. This is mainly due to the weight reduction that can be achieved using foams and for their mechanical energy absorption and acoustic damping capabilities. An accurate knowledge of the mechanical behavior of these materials, especially under dynamic loadings, is thus necessary. Unfortunately, metal foams and in general “soft” materials exhibit a series of peculiarities that make difficult the adoption of standard testing techniques for their high strain-rate characterization. This paper presents an innovative apparatus, where high strain-rate tests of metal foams or other soft materials can be performed by exploiting the operating principle of the Hopkinson bar methods. Using the pre-stress method to generate directly a long compression pulse (compared with traditional SHPB), a displacement of about 20 mm can be applied to the specimen with a single propagating wave, suitable for evaluating the whole stress-strain curve of medium-sized cell foams (pores of about 1–2 mm). The potential of this testing rig is shown in the characterization of a closed-cell aluminum foam, where all the above features are amply demonstrated.

## 1. Introduction

For more than 20 years, metallic and particularly aluminum alloy foams have become an interesting material class especially in aerospace and automotive industries [1,2]. High-energy absorption capabilities and lightweight make these materials highly attractive compared with traditional ones like bulk metals or polymers. This trend is also promoted by European Directives concerning environment sustainability (reduction of polluting emissions) and safety for all road users. In the security area, foams are also potential candidate materials for mitigating the effects of blast loads on structures [3]. From the scientific point of view, the material poses several challenges and many authors have engaged in the investigation of the mechanical behavior of metallic foams: inhomogeneous structure, complex nonlinear behavior, strain-rate, density, and hydrostatic stress tensor sensitivity are fundamental issues that influence foam behavior in different loading conditions [4,5].

The effect of density appears to be well-established [2,3,4,5] with higher densities resulting in higher strengths. However, this is not the case for the strain-rate sensitivity, where it is possible to find in literature apparently inconclusive experimental data concerning even the same foam base material. This is because the cell size and the foam morphology are important parameters defining its mechanical properties, and most likely because no standardization exists in high strain-rate tests. Thus, particular problems can arise in this kind of testing due to the low acoustic impedance and strength of this material class.

Both strain-rate sensitivity and absence of it have been observed in a variety of different foams. For Alporas closed-cell foams, an apparent strain hardening and more than 50% enhancement of the quasi-static plateau strength are reported in references [6,7,8,9] for compression rates greater than 1000/s. On the contrary, for Alcan2 (open-cell) foams and for compression rates up to 1000/s it is reported in reference [10] that the specific energy absorption remains almost invariant. Duocel (open-cell) and Alulight (closed-cell) foams are reported to exhibit a similar rate insensitive behavior [11,12,13] with respect to their plateau strength for strain rates exceeding 1000/s. The comprehensive experimental study of closed-cell Hydro/Cymat aluminum foam in references [14,15] shows that the plastic collapse strength of the foam changes appreciably with compression rate for the two cell sizes of 4 and 14 mm. It is also attempted in reference [14] to establish a consistent framework in terms of foam failure mechanisms and careful definition of the main parameters (plateau stress and densification strain) capable of identifying and explaining the seemingly conflicting conclusions between nominal strain-rate and dynamic strength.

The current work attempts to contribute to overcome the above-mentioned inconsistencies through improvements of the testing technique. An innovative apparatus is introduced, specifically designed to perform compressive high strain-rate tests on metal foams or other soft materials by exploiting the operating principle of the Hopkinson bar methods. As recalled, the conventional SHPB (Split Hopkinson Pressure Bar) produces relatively short duration incident pulses (less than 0.3 ms), thus small displacements, and consequently it is suitable for small-sized homogeneous specimens (millimeter order of magnitude), where even small displacements can result in appreciable strains. However, foams are inhomogeneous materials and in general require larger specimens (centimeter order of magnitude) in order for the mechanical tests to yield meaningful results. The current apparatus employs a pre-stressed bar method (instead of the striker bar of the conventional SHPB) to generate directly a long compression pulse (≈3 ms); a displacement of about 20 mm can thus be applied to the specimen with a single propagating wave, suitable for evaluating the whole stress-strain curve of medium-sized cell foams (pores of about 1–2 mm). To demonstrate the potential of this testing rig, the characterization of closed-cell foams—manufactured by Aluinvent (Felsőzsolca, Hungary)—has been carried out and the results obtained are shown and discussed.

## 2. Modified Hopkinson Pressure Bar for Soft Materials (MHPB-SM)

As mentioned before, aluminum foams and soft materials in general exhibit some particular problems during high strain-rate mechanical testing, especially by means of Hopkinson bar techniques. The main issues are as follows:

- Low signal amplitude to noise ratio. This phenomenon is essentially due to the low strength of tested material compared with the sensitivity of the equipment bars. This aspect substantially decreases the accuracy of the data obtained with Hopkinson bar techniques.
- If solid steel bars are employed, the strong impedance mismatch between specimen and equipment bar makes sometimes difficult to interpret the experimental data concerning this type of test.
- The use of plastic bars in SHPB to obtain stronger strain signals inevitably introduces other problems (marked wave dispersion due to high internal damping and non-linear elastic behavior, *etc*.) that dramatically increase the complexity of data processing [16].
- It is normally difficult to examine the whole stress-strain curve of this kind of materials because of their high compliance compared with the small displacement capability of standard Hopkinson bars.
- The specimen sizes imposed by Hopkinson techniques constraints (compared with the foam cells dimensions) are usually too small to have a fully representative material volume.

**Figure 1:**
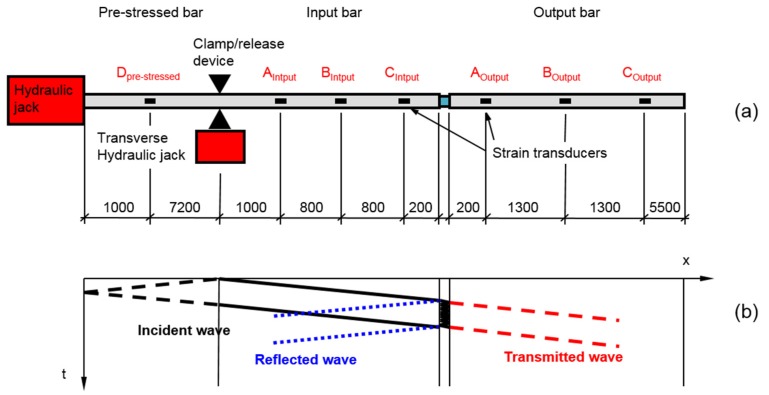
(**a**) Sketch not to scale of Modified Hopkinson Pressure Bar (dimensions in mm); and (**b**) related Lagrangian wave propagation diagram.

To avoid these problems, many researchers have tried to develop alternative, more performant Hopkinson-bar based rigs [4,15,16]. In this direction, at the European Laboratory of Structural Assessment (ELSA) of the Joint Research Centre a new Hopkinson-bar based equipment has been developed capable of performing compressive tests on medium cell-size materials. The new rig can be defined as a modified Hopkinson pressure bar for soft materials (MHPB-SM) and its operation principle can be deduced from the sketch in Figure 1. As in the standard MHPB [17], to increase the input pulse wavelength and consequently the maximum displacement applied to the specimen, the pulse is generated by pre-stressing a long portion of the input bar and instantly releasing it with a special clamping device. At this point, the test carries on as in the standard Hopkinson bar, as shown in the Lagrangian propagation diagram of Figure 1.

This technique has mainly been adopted to generate tensile pulses but with some small expedients, it can be successfully used to produce long compression pulses, too. Except for the pulse generation mechanism, a MHPB test is identical to a standard SHPB test, with the advantage of the longer incident wave. Obviously, the adoption of longer waves (and depending on the relative lengths of the pre-stressed, input and output bars) can introduce wave-overlapping problems that have to be efficiently solved in order to correctly reconstruct the forces and displacements applied to the specimen. A more detailed presentation of the apparatus follows below.

### 2.1. Mechanical Structure

**Figure 2:**
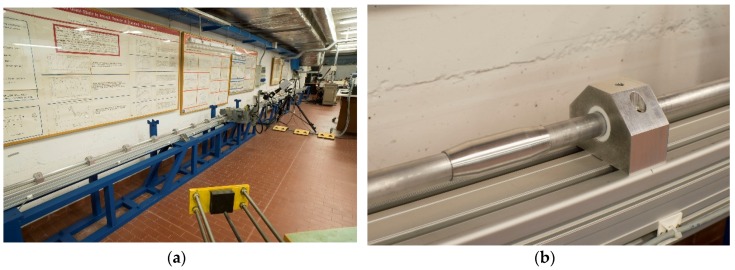
(**a**) MHPB-SM at the ELSA of JRC; (**b**) detail of bar-joint and support.

A principal target of the MHPS-SM is to increase the maximum displacement applied to the specimen using the pre-stressing method. To reach this goal it is necessary to increase the bar’s length taking into account technological problems in the manufacturing of very long bars (more than 6 m) and the increasing cost due to customized products. The technological solution adopted for the MHPB-SM rig is shown in Figure 2.

The MHPB-SM is made of high-strength aluminum (2024 alloy with *σ_y_* ≈ 325 MPa and *E* = 72,000 MPa, *ρ* = 2700 kg/m^3^) bars of 20 mm diameter and of a maximum length of 3 m, which guarantee a simple machining of the bar-ends and, consequently, low manufacturing costs. The bar diameter has been chosen to match completely with the specimen sizes adopted. Several bars are connected to assemble the final input and output bars using properly shaped bar-ends and sleeves in order to limit spurious reflections during the wave propagation. Using this concept, a single, uniform input bar of 11.0 m (with a pre-stressed part of 8.2 m and an incident part of 2.8 m) and an output bar of 8.3 m have been assembled for a total apparatus length of about 19 m. This configuration allows a compression pulse of almost 3 ms (≈2 × 8.2 m / 5150 m/s) to be generated and consequently a displacement of more than 15 mm to be applied to the specimen.

The bars are supported using low-friction Teflon bushings mounted in aluminum supports as shown in Figure 2b and the initial pre-stressing is provided using an oleo-dynamic high-pressure jack. Obviously, to avoid elastic-buckling phenomena, the distance between the supports has been carefully designed in function with the envisaged maximum test pre-compression.

The high velocity clamp/release device (the so called “*θ*-clamp”) consists of several high-strength steel components (Maraging steel) loaded by a transverse high-pressure hydraulic jack, which clamps the bar by means of friction using an expendable fragile bolt to transfer forces. The clamp position is fixed, and a test takes place as follows:

- The input bar is clamped with the *θ*-clamp by applying an adequate force with the transverse hydraulic jack;
- The pre-stressed part is next compressed by using the main hydraulic jack at the bar left end;
- Having reached the design pre-compression (it depends on the test desired input pulse) in the pre-stressed part, the fragile bolt of the *θ*-clamp is forced to break (by further increasing the transverse jack load);
- The pre-stressed bar is then rapidly released thus generating a compression pulse with a rise time of about 20 µs, which starts propagating in the incident bar towards the specimen.

It must be underlined that the intrinsic modularity of this equipment design allows changes in the bar configuration (for what concerns length and diameter) to be performed easily without substantial mechanical interventions. This feature may be important, especially in Hopkinson-based tests where the equipment must be “tuned” on the specimen that has to be tested. Thus, further developments of the equipment could increase the tested specimen sizes to 40–50 mm of diameter with a maximum displacement capability of about 40–50 mm.

### 2.2. Instrumentation

Another fundamental component of the MHPB-SM is certainly the instrumentation adopted during the tests, which is mainly composed of two elements: a standard strain-gage-based measurement system and a high-speed camera. These two independent devices allow a greater comprehension of the dynamic phenomena that cannot be directly observed, and guarantee more accurate results due to the data crosschecking.

**Figure 3:**
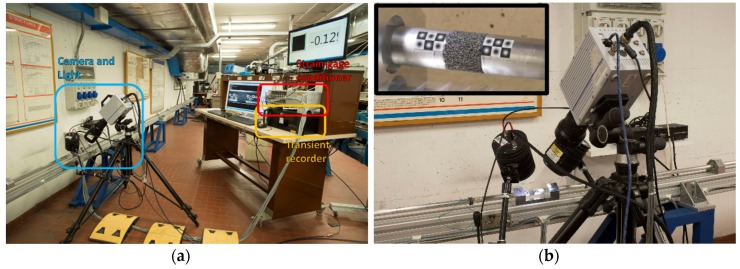
(**a**) Instrumentation adopted in MHPB-SM tests; (**b**) detail of the high-speed camera, specimen, and optical targets (detail on the upper left corner).

The strain-gage measurement system employed is composed of a series of semiconductor strain-gages, a high-speed conditioning device (with a cut-off frequency of more than 500 kHz) and a transient recorder (with a sample rate of at least 10 MSample/s), as shown in Figure 3a. The semiconductor strain-gages have the advantage of a higher gage factor compared with standard strain-gages (60 times greater), a fact that allows aluminum bars to be adopted for the characterization of soft materials without any compromise in what concerns the signal-to-noise ratio. A small drawback of these sensors lies in their reduced linear operation range compared with the standard strain-gages. However, this feature can be simply evaluated, and eventually compensated, by statically calibrating the sensors in terms of force, using a reference load-cell, as described in the next paragraph. The strain measurement stations, located as reported in Figure 1, have a half-bridge circuit setup, with two strain-gages placed longitudinally on the bar (one gage diametrically opposite to the other). This configuration allows the measurement sensitivity to be doubled, and simultaneously the compensation of infinitesimal bar bending.

For what concerns the optical instrumentation (Figure 3b), in all tests a high-speed photo sequence has been acquired at a frame rate of about 25,000 fps. At this velocity, it is essential to have available a strong light source in order to reduce the exposure time and acquire “frozen” images. Thus, a LED light source has been used that strobed at the same frequency as the camera frame-rate. The cold feature of the LED light ensures negligible thermal effects on the mechanical properties of the tested material even after long exposures or in cases of high thermal sensitivity (as occurs in plastic foams). Special black and white Gaussian targets have also been placed on the bars in contact with the specimen, to be used for applying an accurate optical tracking algorithm.

### 2.3. Data Elaboration

One of the critical aspects of MHPB-SM concerns the data elaboration of the recorded signals. The long propagating pulse, compared with the length of the adopted bars, makes essential the implementation of an appropriate algorithm for separating correctly overlapped waves that travel simultaneously in opposite directions during the test. In fact, depending on the relative lengths of the pre-stressed, input, and output bars, at the strain-gage stations in the MHPB-SM incident, reflected and transmitted waves are not separated as in the classical SHPB. Of the several algorithms found in the literature, fully satisfactory results have been obtained when using the algorithm proposed in reference [18]. This technique exploits the exact formulation of three-dimensional wave propagation theory in infinite elastic rods and allows the accurate reconstruction of the strain history at any bar point starting from at least two independent strain measurements. Other approximate and iterative algorithms, as for example in reference [19], do not guarantee the required accuracy for the whole recorded time due to error propagation after a few iterations. However, the wave separation method adopted needs some preliminary steps to tune certain mathematical stabilization coefficients in order to achieve the best results, as will be presented in the following.

For what concerns the optical analysis of the high-speed photo sequence, two different algorithms have been applied: a fast computational tracking algorithm and a full field procedure, called the optical flow method. The fast computational tracking algorithm [20] exploits the black and white Gaussian targets mentioned before and allows the accurate evaluation of target displacements with an accuracy of about 1 µm (for an image pixel dimension equal to about 0.1 mm) with very low computational costs. Data produced with this numerical procedure can be directly compared to the data obtained from the Hopkinson bar analysis of the strain-gage measurements in order to assess the effectiveness of proposed elaboration. On the other hand, the Optical Flow method [21] is suitable for evaluating the displacement field of the specimen during the test. Even though the accuracy in terms of evaluated displacements is substantially coarser compared with the previous method and needs greater computational effort, this algorithm can supply useful information and insight concerning the specimen collapse mode.

## 3. Experimental Tests and Data Analysis

Due to its peculiar features, before proceeding with the compressive characterization of foam specimens, the MHPB-SM requires a series of experimental steps to tune the data elaboration procedures in order to achieve optimized results. First, it is essential to verify the strain measurement chain with regards to repeatability, accuracy, and linearity. This check is performed statically in terms of force using a reference load cell placed between the input and the output bars and compressing them with the pre-stressing actuator. All the strain-gage measurement points and the reference load cell are in series and they must measure the same force (except for the friction between the bars and the Teflon bushings).

**Figure 4:**
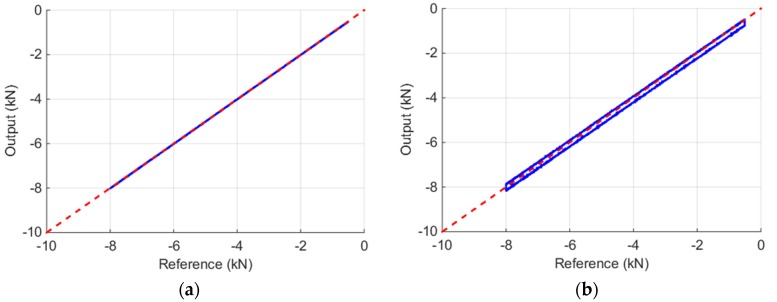
Calibration of measurement point on: (**a**) the instrumented bar segment (measurement point A_input_); and (**b**) in the proximity of the pre-stressing jack (measurement point D).

Figure 4 shows the calibration curves for two measurement points on the apparatus bars. Figure 4a displays three consecutive loading and unloading paths recorded at one of the measurement points (*A_input_*) on the instrumented input bar segment farther away from the specimen. As can be seen, in the tested force range there is no non-linearity present, and friction phenomena are negligible. However, in the data obtained from a measurement point placed near the pre-stressing jack (Figure 4b) friction is present. It is quantifiable to approximately 0.3 kN in the calibration range and it is due to the 30 supports between the reference load cell and the measurement point. This fact further justifies the instrumentation of the bar segments in the proximity of the specimen. In addition, in the calibration step it is possible to verify and, if necessary, adjust with a correction coefficient the experimental results in order to ensure the same sensitivity for all measurement points (different sensitivities occur due to strain-gage misalignments or different amplifier gains).

This last issue is an essential requirement of the separation algorithm presented before, as pointed out in [18].

Finally, it must be underlined that, the MHPB-SM has the additional advantage, compared with other Hopkinson-bar based apparatuses, to be easily calibrated using the pre-stressing jack already mounted. In fact, for the calibration, it is not as simple to load all the bar strain transducers simultaneously by generating the compression pulse with an impacting striker bar in the SHPB [22] or by using the tensile pre-tensioning method adopted in [23]. A robust calibration procedure is an essential requirement to ISO 9001 quality standard [24] of a modern material testing equipment.

### 3.1. Void Test

The next essential step towards “tuning” the data elaboration procedure in order to enhance the accuracy of the data analysis is the so-called “void test”: in practice, the input and output bars are brought in contact, a compression test without a specimen is performed, and the forces and displacements at the input-output bar interfaces are reconstructed and compared. This test can be considered as representing a well-known boundary condition problem, and is in particular useful for tuning some stabilization coefficients of the wave separation algorithm proposed in reference [18].

**Figure 5:**
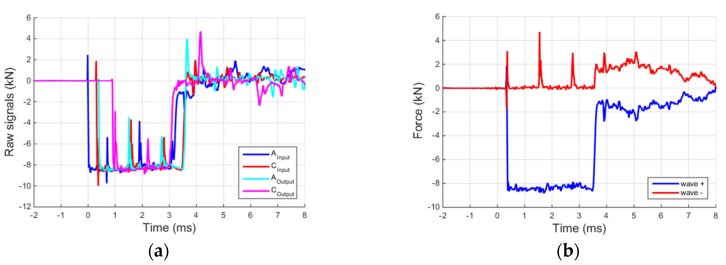
(**a**) Raw signals recorded on the Input and Output bars on two locations; and (**b**) wave propagating in the input bar reconstructed at the input–output bar interface.

As mentioned in the previous paragraph, starting from at least two strain-histories recorded in different locations on a single bar (Figure 5a) it is possible to reconstruct the ascending and descending waves in any other bar location using the deconvolution algorithm [19]. The algorithm uses the theoretical three-dimensional wave propagation theory in infinite elastic rods (Pochhammer-Chree theory), and works in the frequency domain. It further requires the prior determination of some stabilization coefficients, which is accomplished by using the recorded data sets mentioned above. Figure 5b shows the results for the input bar and in particular the ascending (wave +) and descending waves (wave −), respectively, at the input-output bar interface. The adopted algorithm has the advantage of working on the entire time domain and of directly applying the correct time shift to the reconstructed waves. As seen in Figure 5b, the algorithm is also able to satisfactorily identify spurious reflections due to the small impedance mismatch at the input-output bar interface (first peak on the reflected wave) or at the bar sleeve-joints (second and third peak on the reflected wave).

**Figure 6:**
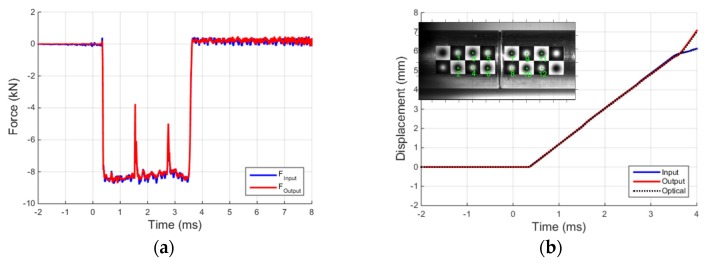
(**a**) Equilibrium check at input-output bar interface; and (**b**) comparison between displacements obtained with Hopkinson bar theory and with the fast tracking optical algorithm.

At this point, by applying the well-known Hopkinson bar formulae, the forces and displacements of the two bars at their interface can be reconstructed, as shown in Figure 6. In particular, the forces at a bar interfaces are proportional to the sum of the two ascending and descending waves previously computed, whereas the displacements are proportional to the difference of the same waves. Figure 6a presents the equilibrium check at the input-output bar interface in the time domain, and the excellent quality of the results is to be attributed to the previous calibration and the precision tuning of the stabilization coefficients of the separation algorithm. With reference to Figure 6, it can be noted that the two bars remain in contact for about 3.2 ms (the duration of the input pulse) and then the output bar moves away from the input bar (and the interaction force drops to zero). Figure 6b shows the comparison between the input and output bar end displacements obtained with the Hopkinson bar theory and with the fast tracking optical algorithm, described in the previous paragraph. To analytically evaluate the differences between the curves reported in Figure 6b (two “Hopkinson” and two “optical” displacement data series) the relative error is determined, defined as:

(1) $$e_{\%}=\frac{\sum^{\text{​}}\left|D_{Hopkinson}-D_{Optical}\right|}{\sum^{\text{​}}\left|D_{Optical}\right|}\times 100$$

where DHopkinson and DOptical are, respectively, the bar end displacements obtained with the advanced Hopkinson elaboration and the optical algorithms, previously mentioned. This index can be thought as representing the mean error divided by the reference mean displacement. Relative errors of 0.88% and 0.22% have been computed for the input and the output bars, respectively.

The optical displacement reported in the graph is the average displacement of the targets adjacent to bar interface (targets 5,6 for the input bar and 7,8 for the output bar). The three curves of Figure 6b, based on two independent measurement data sets, are practically coinciding up to about 4 ms, thus demonstrating once again the accuracy of the data processing implemented. After the detachment of the two bars, some oscillations reduce the accuracy of the Hopkinson bar elaboration (especially for the input bar). However, this can be readily identified and controlled thanks to the high-speed camera measurements, and in no way would it influence the outcome of compression tests (already completed by that time).

### 3.2. Foam Tests

**Figure 7:**
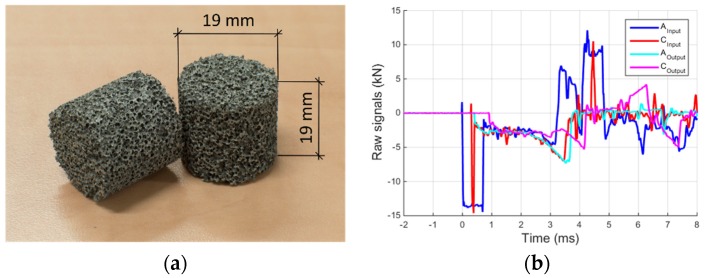
(**a**) Specimen of aluminum foam ALUHAB; and (**b**) raw signals recorded on the input and output bars on two locations.

Following the static calibration of the strain transducers and the dynamic tuning of separation algorithm achieved with the void test, it is straightforward to apply the MHPB-SM technique to an actual compression test on foamed material. A series of such compression tests have been performed on cylindrical specimens with a diameter of 19 mm and a length of 19 mm (Figure 7a).

The specimens have been cut using electrical discharge machining from a block of ALUHAB aluminum foam with a density of about 0.55 g/cm^3^ and an average pore size of 1 mm, manufactured by Aluinvent. This cell size renders the specimen of 19 mm size suitable for the characterization of the foam with the MHPB-SM, as it constitutes a representative volume of the material. The ALUHAB aluminum foam comes under the market name of EN43100 8ALO2, which is manufactured from the aluminum alloy EN43100 (AlSi10Mg base) and where 8ALO2 means 8% in volume of Al_2_O_3_ particles of 2 microns. The technology uses special high temperature admixing to homogeneously disperse the particles and thus creates a stable, foamable aluminum melt. This technology permits the injection of optimally sized bubbles from 10 to 0.5 mm range into the melt.

**Figure 8:**
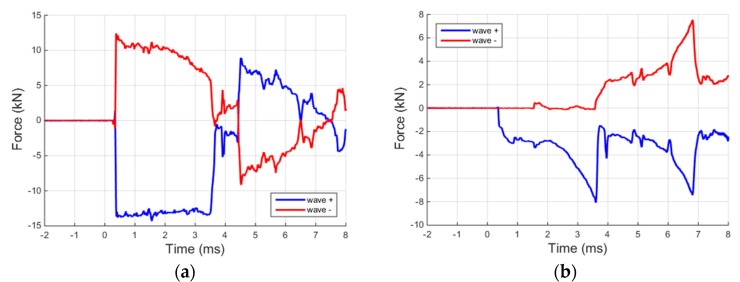
Waves propagating in the (**a**) input; and (**b**) output bars, reconstructed at the interfaces with the specimen.

As in the void test, the raw signals recorded during the material tests are totally overlapped for both input and output bars, as shown in Figure 7b. Applying the separation algorithm on the two data series related, respectively, to the input and output bars, the ascending and descending waves are accurately reconstructed, Figure 8. Figure 8a shows clearly the rectangular incident and the reflected waves. After about 4 ms, the reflected wave comes back to the bar end (previously in contact with the specimen) and continues to travel inside the input/pre-stressed bar generating the strange wave tails. On the other hand, Figure 8b shows the two waves travelling in the output bar. For about 3.2 ms the ascending wave is practically equal to the force applied to the specimen except for the small reflections due to the bar joints (small oscillations of the descending wave in the initial 3.2 ms).

**Figure 9:**
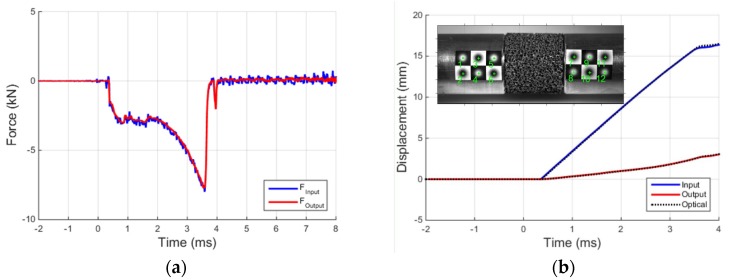
(**a**) Equilibrium check at specimen-bars interfaces; and (**b**) comparison between displacements obtained with Hopkinson bar theory and with the fast tracking optical algorithm.

At this point, the forces and displacements applied to the two faces of the specimen can be determined. In this way, the specimen force equilibrium is checked and successfully verified, as shown in Figure 9a. It is interesting to underline that in all graphs presented no other filtering has been applied in order to be able to focus on and assess only the performance of the equipment and the data elaboration procedure. Although the input force is noisier compared with the output one (which is naturally filtered by the specimen mass and damping), the specimen equilibrium is attained even when the specimen is crossed by the ascending profile of the compression pulse. For what concerns the displacements, also in this case, there is an excellent agreement between the data derived from the strain-gage measurements and those of the high-speed photo sequence (Figure 9b). The absence of delays between the two data series once again validates the effectiveness of the applied data elaboration. The relative errors computed using Equation (1) are, respectively, 0.88% and 1.03% for the input and the output bar.

Based on the above and the hypothesis of uniform stress-strain fields in the specimen below, the histories of stress *σ*, strain-rate $\dot{\varepsilon }$, and strain *ε* of the specimen can be written using the standard Hopkinson bar relationships:

(2) $$\sigma \left(t\right)=\frac{F_{i}+F_{o}}{2\;A_{s}}=\frac{E\;A_{b}}{2\;A_{s}}\left(\varepsilon_{+\;i}\left(t\right)+\varepsilon_{-\;i}\left(t\right)+\varepsilon_{+\;o}\left(t\right)+\varepsilon_{-\;o}\left(t\right)\right)$$

(3) $$\dot{\varepsilon }\left(t\right)=\frac{v_{i}-v_{o}}{L}=\frac{C_{0}}{L}\left(\varepsilon_{+\;i}\left(t\right)-\varepsilon_{-\;i}\left(t\right)-\varepsilon_{+\;o}\left(t\right)+\varepsilon_{-\;o}\left(t\right)\right)$$

(4) $$\varepsilon \left(t\right)=\int^{\text{​}}\dot{\varepsilon }\left(t\right)dt=\frac{C_{0}}{L}\int^{\text{​}}\left(\varepsilon_{+\;i}\left(t\right)-\varepsilon_{-\;i}\left(t\right)-\varepsilon_{+\;o}\left(t\right)+\varepsilon_{-\;o}\left(t\right)\right)dt$$

where, *F* and *v* are, respectively, the forces applied and the velocities at the two specimen surfaces (*i* = input, *o* = output); *A_b_* and *A_s_* the bar and the specimen cross-sections; *L* the specimen length; and *ε_+_* and *ε_−_* are respectively the ascending and descending strain waves reconstructed at the specimen-bar interfaces (*i* = input, *o* = output), derived from Figure 8.

**Figure 10:**
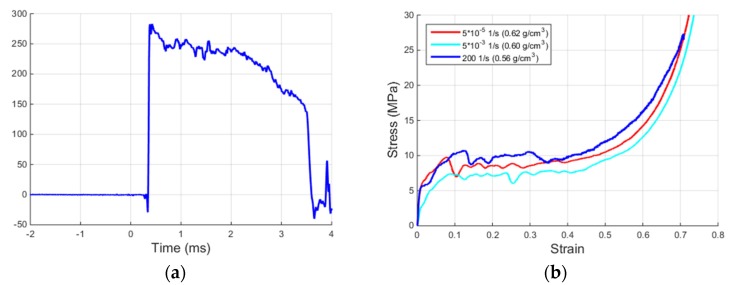
(**a**) Specimen strain-rate during a MHPB-SM test; and (**b**) engineering stress-strain curve of ALUHAB foam.

The two graphs in Figure 10 show the specimen strain-rate during a MHPB-SM test and the specimen stress-strain curve obtained using relations Equations (2)–(4). It is observed that, thanks to the characteristic, well-pronounced plateau in the stress-strain curve, the test is performed at an almost constant strain-rate of approximately 200 s^−1^. This aspect is important if the material strain-rate sensitivity is to be investigated; clearly, other strain-rates can be produced by changing the initial compression force in the pre-stressed bar and/or by varying the length of the specimen. Concerning the apparatus limits in the current configuration, it is evident that they are connected to the magnitude of the compressive force in the pre-stressed part. This force must be such that the bar remains always elastic, it must be less than the buckling load, and it must not exceed the capacity of the clamp/release mechanism. Specifically, a pre-stress between 15 and 40 kN can be easily applied, which would correspond, respectively, to a specimen strain-rate between 100 and 400 1/s (assuming the same specimen strength). By halving the specimen length (and still maintaining a representative material volume), these strain-rate values would be almost doubled, as Equation (3) indicates. Also for the same specimen length, larger pre-loads would produce larger maximum deformations (full densification with 40 kN pre-stress and only part of it with 15 kN pre-stress).

With reference to the stress-strain curve of Figure 10b, it is noticed that the strain reached when using a specimen of larger dimensions (than that for metals or plastics of the standard SHPB) is more than 70%. This feature allows an effective characterization of the stress-strain curve of foamed and, in general, soft materials. In fact, apart from the stress plateau, which is entirely reproduced, even the onset of the specimen densification phase is well captured. A shorter specimen would have allowed its full reproduction. For comparison purposes, two low strain-rate stress-strain curves of this foam (obtained with a servo-hydraulic universal testing machine) are also included in Figure 10b. For the strain-rate range considered, the strain-rate sensitivity appears to be negligible, especially when compared with the natural data scattering of this type of material.

**Figure 11:**
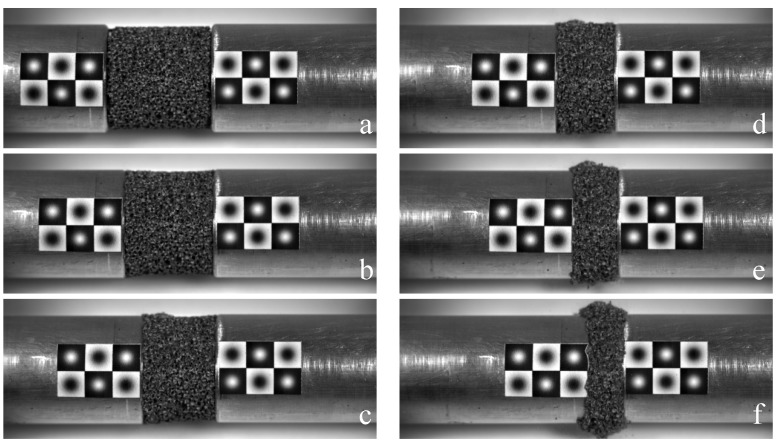
High-speed photo sequence of a MHPB-SM test: (**a**) 0.36 ms; (**b**) 1.00 ms; (**c**) 1.64 ms; (**d**) 2.28 ms; (**e**) 2.92 ms; (**f**) 3.56 ms.

Finally, Figure 11 shows an example of a series of a high-speed photo sequence frames recorded during a MHPB-SM test on an aluminum foam specimen. As mentioned before, in addition to the fast tracking algorithm useful to measure the bar’s displacements, it is possible to apply the optical flow method to evaluate the displacements and eventually the strain field of the specimen during the test. This type of analysis provides the possibility to study the collapse mechanism of a foamed material and to check the existence of peculiar instability phenomena that can occur under dynamic conditions.

## 4. Conclusions

The work presents an innovative testing apparatus for performing dynamic compression tests on a class of materials, which includes the metal foams and in general soft materials. Such materials are widely adopted in modern structural engineering for their excellent performance in terms of dissipated energy during impact, damping, and light weight. As explained, the dynamic characterization of these materials is not trivial due to peculiar problems strictly related to their physical structure, and it needs special equipment developed ad hoc in order to effectively reach this goal.

The salient features of this MHPB-SM can be summarized as follows: The apparatus has been designed with a modular mechanical structure able to easily undergo modifications regarding equipment geometry (bar dimensions) or test requirements (increase of the maximum force or strain capabilities). Thanks to the semiconductor strain-gages adopted, the signal to noise ratio has been substantially increased. The aluminum bars used reduce the impedance mismatch with the specimen (compared to the standard high-strength steel bars), and in addition they obviate nonlinearity and strong damping phenomena that plastic bars would have caused. Of great advantage with respect to the conventional SHPB, long pulses are generated in the MHPB-SM and the specimen is acted upon for an equally long time. Currently this of the order of 3 ms. The amplitude of these rectangular pulses can be readily adjusted by changing the compression of the pre-stressed part. The state-of-the-art instrumentation and data elaboration employed allow the efficient reconstruction of these very long compression pulses. Thus, the full material stress-strain curve can be efficiently investigated using relatively large specimens (up to 20 mm of diameter and length). These dimensions guarantee a representative material volume with foams of pore sizes up to 2 mm.

Void tests and tests with aluminum foam specimens of an average pore size of 1 mm have been performed. The reported high-quality testing results fully demonstrate the capabilities of the apparatus and of the procedures employed.

Further steps in in the apparatus development would be to increase the bar diameter and the total apparatus length in order to characterize larger pore foams which require specimens with a representative volume of about 50 mm of diameter and length. Finally, it has been seen that the employment of a high-speed camera and image analysis with quantitative optical algorithms give the possibility of comparing classical strain-gage data with a series of independent and accurate displacement measurements. This feature increases the accuracy and reliability of the results and aids effectively in the comprehension of dynamic phenomena taking place.
